# Structure-based enzyme engineering improves donor-substrate recognition of *Arabidopsis thaliana* glycosyltransferases

**DOI:** 10.1042/BCJ20200477

**Published:** 2020-08-07

**Authors:** Aishat Akere, Serena H. Chen, Xiaohan Liu, Yanger Chen, Sarath Chandra Dantu, Alessandro Pandini, Debsindhu Bhowmik, Shozeb Haider

**Affiliations:** 1 Pharmaceutical and Biological Chemistry, UCL School of Pharmacy, London WC1N 1AX, U.K.; 2 Computational Sciences and Engineering Division, Oak Ridge National Laboratory, Oak Ridge, TN 37830, U.S.A.; 3 College of Life Science, Sichuan Agricultural University, Ya'an, China; 4 Department of Computer Science, Brunel University London, Uxbridge UB8 3PH, U.K.

**Keywords:** deep learning, glycosyltransferases, mass spectrometry, molecular dynamics

## Abstract

Glycosylation of secondary metabolites involves plant UDP-dependent glycosyltransferases (UGTs). UGTs have shown promise as catalysts in the synthesis of glycosides for medical treatment. However, limited understanding at the molecular level due to insufficient biochemical and structural information has hindered potential applications of most of these UGTs. In the absence of experimental crystal structures, we employed advanced molecular modeling and simulations in conjunction with biochemical characterization to design a workflow to study five Group H *Arabidopsis thaliana* (76E1, 76E2, 76E4, 76E5, 76D1) UGTs. Based on our rational structural manipulation and analysis, we identified key amino acids (P129 in 76D1; D374 in 76E2; K275 in 76E4), which when mutated improved donor substrate recognition than wildtype UGTs. Molecular dynamics simulations and deep learning analysis identified structural differences, which drive substrate preferences. The design of these UGTs with broader substrate specificity may play important role in biotechnological and industrial applications. These findings can also serve as basis to study other plant UGTs and thereby advancing UGT enzyme engineering.

## Introduction

Glycosyltransferases (GTs) are a large family of structurally conserved enzymes responsible for catalyzing the transfer of a sugar moiety from an activated donor sugar to an acceptor molecule [1]. The sugar transfer reactions, known as glycosylation, is quantitatively the most important reaction on earth as they account for the biosynthesis of most of the biomass [2]. Glycosylation of plant secondary metabolites is catalyzed by uridine diphosphate (UDP) glycosyltransferases (UGTs) belonging to family 1 GTs [3]. These plant secondary metabolites include alkaloids, terpenoids, flavonoids, isoflavonoids, and other small molecules. UDP activated sugars act as donors in the glycosylation reaction with UDP-glucose (UDP-Glc) serving as the primary sugar donor, followed by UDP-galactose (UDP-Gal), UDP-rhamnose, UDP-xylose, and UDP-glucuronic acid, respectively [4]. Furthermore, plant-derived natural products, as therapeutics of human health are of considerable interest to pharmaceutical industry [5]. For example, the planet-produced enzymes like ELELYSO (Protalix BioTherapeutics), is central to the production of lysosomal human GBA enzyme in carrot cells. Another human lysosomal hydrolase is α-galactosidase A, used for treating Fabry disease [5]. As of today, there are a total of 122 *Arabidopsis thaliana* UGT genes, which are grouped into 14 phylogenetic groups A-N (Supplementary Figure S1) [6].

All crystal structures of plant UGTs (Supplementary Table S1) show that the enzymes adopt the GT-B fold, which consists of two Rossmann-like domains; the C- and N-terminal domains [7–17]. A cleft located in between the two domains bind substrates (Supplementary Figure S2**)**. The N-terminal domain is more variable and can accommodate diverse acceptors while the more conserved C-terminal domain binds nucleotide sugar donors [1,17].

A conserved 44 amino acid motif found in the C-terminal domain in all UGTs called the PSPG (Plant Secondary Product GTs) box (Supplementary Figure S3) is regarded as the ‘consensus sequence’ and considered a characteristic structural feature of plant UGTs [18,19]. The consensus sequence is routinely used in bioinformatics studies to identify plant GTs from various databases [20]. The PSPG box is involved in the binding of the nucleotide sugar UDP moiety and offers bulk of the sugar donor interactions [6].

The linker region connecting the two domains varies in length and sequence. However, the C-terminal part of the linker is usually positioned around the uridine part of the UDP-donor sugar in many plant UGTs. Upon donor sugar binding, the linker loop goes through a conformational change. This is consistent with the suggestion of the region's ability to adapt conformational changes upon sugar binding [11].

Several loop regions in plant UGT structures (besides the PSPG motif residues) offer critical interactions with either or both substrates (Supplementary Figure S4A). An example of such loop region is the C-terminal C1 loop, which interacts with the donor sugar. Across all 10 plant UGT crystal structures that are available, the length of this loop is highly conserved except in UGT71G1 which has an extra residue (Supplementary Figure S4B). The loop also contains a conserved glycine next to a serine/threonine, which directly binds to the β-phosphate part of sugar [8,10,12,13].

For most plant UGTs, their *in vivo* and *in vitro* functions are still unknown [21]. From *A. thaliana*, only two crystal structures of UGTs are available out of the 122 genes that have been identified (http://www.p450.kvl.dk/). In addition, more than half of these UGTs are yet to be biochemically characterized. To gain better insight into UDP-sugar specificity mechanism, there is a need for crystal structures of UGTs complexed with substrates [1]. However, in the absence of the X-ray structures, comparative homology models are excellent tools to study the structure-function of proteins [22,23]. Several previous studies have reported UGTs structural manipulation to engineer mutants with improved sugar substrate recognition [24–27]. Homology modeling, docking, and mutation studies in *Pf*UGT88D7 pointed to R350 and S127 as important residues for UDP-glucuronic acid binding. Both mutants R350W and S127 T showed decreased glucuronosyl and increased glucosyl activity [25]. Molecular modeling on UDP-glucuronosyltransferase *Bp*UGT94B1 identified an N-terminal residue R25 to be crucial and specific for the enzyme activity with UDP-glucuronic acid [24]. Similar manipulations have also been done around the acceptor binding pocket regions, affecting UGT enzyme activity and altering substrate specificity. While UGT71G1 glucosylated flavonoid genistein at the 7-OH position, its mutant Y202A was able to add sugar at an additional position, 5-OH; giving two products, 7-*O*-glucoside and 5-*O*-glucoside [27]. These mutations, all rationally designed, resulted in the development of new UGTs with enhanced activity. Thus, structurally engineered UGTs may be exploited for enzymatic synthesis of glycosides [26].

In this study, we report on the qualitative substrate specificities of 76E1, 76E2, 76E4, 76E5, and 76D1 Group H *A. thaliana* UGTs using mass spectrometry (MS) based methods. We then construct comparative homology models, carry out all-atom molecular dynamics (MD) simulations in explicit solvent, and employ convolutional variational autoencoder (CVAE) based deep learning analysis to further understand their substrate preferences and identify key amino acid residues involved. Making inferences from the structure–specificity relationship, we rationally designed mutant UGTs, which not only displayed improved donor recognition but also highlighted the functional roles of the mutated residues.

## Materials and methods

### Site-directed mutagenesis

Primer design was done using NEBaseChanger v1.2.7 (http://nebasechanger.neb.com/) and all primers produced by Eurofin Genomics Ltd. Templates DNAs (wildtypes 76D1, 76E2, and 76E4) were extracted via QIAprep Spin miniprep kit protocol. Q5 Site-directed mutagenesis kit protocol (from NewEngland Biolabs) was used to make mutants from the wildtype template DNAs. Mutagenesis was done in three stages of exponential amplification (polymerase chain reaction), digestion and transformation. Mutant plasmid DNAs was extracted via miniprep. Mutant DNAs were sequenced (Source Bioscience) to check correct mutations. The primer sequences for 76E2 D374E: forward primer TTTCACCGGGGAGCAGAAAGTCA, reverse primer GGCCTACATATCATCGGAAC; 76D1 P129T: forward primer GGTCTTTAGCACTTCTTCCGCCG, reverse primer ATCTTTGGCAGATTCATATCTTCCG; 76E4 K275L: forward primer CTTGGGAACCTTAGCTCACATGG, reverse primer CTTATGTATATGACTGACCTTG and 76D1 G347C: forward primer TTGGAACCATTGCGGATGGAACTCGTG, reverse primer AACCCTCCCACTGCTCTA.

### Expression and purification of mutant UGTs

Recombinant plasmids were transformed into BL1 (DE3) cells for protein expression. They were then verified via Sanger DNA sequencing (Source Bioscience Ltd). The bacterial cells were grown as previously reported [28]. The GST-tagged recombinant protein was purified by affinity chromatography (columns) and quantified by the Bradford assay. Purified mutant proteins were analyzed on sodium dodecyl sulfate-polyacrylamide gel electrophoresis (SDS–PAGE) gels to verify protein production.

### *In vitro* UGT reaction assay

The UGT enzyme assay included the following components: 1 mM Tris, 1 mM MgCl_2_ (pH 8.0), 10 mM UDP-sugars, 10 mM substrates, and purified target proteins. The donor sugar compounds used in the donor screening are UDP-Glc, UDP-Gal, and UDP-N-acetylglucosamine (UDP-GlcNAc). The reaction mixture was incubated at 37°C for 15 h, terminated with acetonitrile and centrifuged to remove proteins. The supernatant was then analyzed with LC–MS. Glycosylated products were identified by their molecular mass, and these target compounds were subsequently fragmented using MS/MS for confirmation [28].

### HPLC–MS/MS

**Figure 1.**
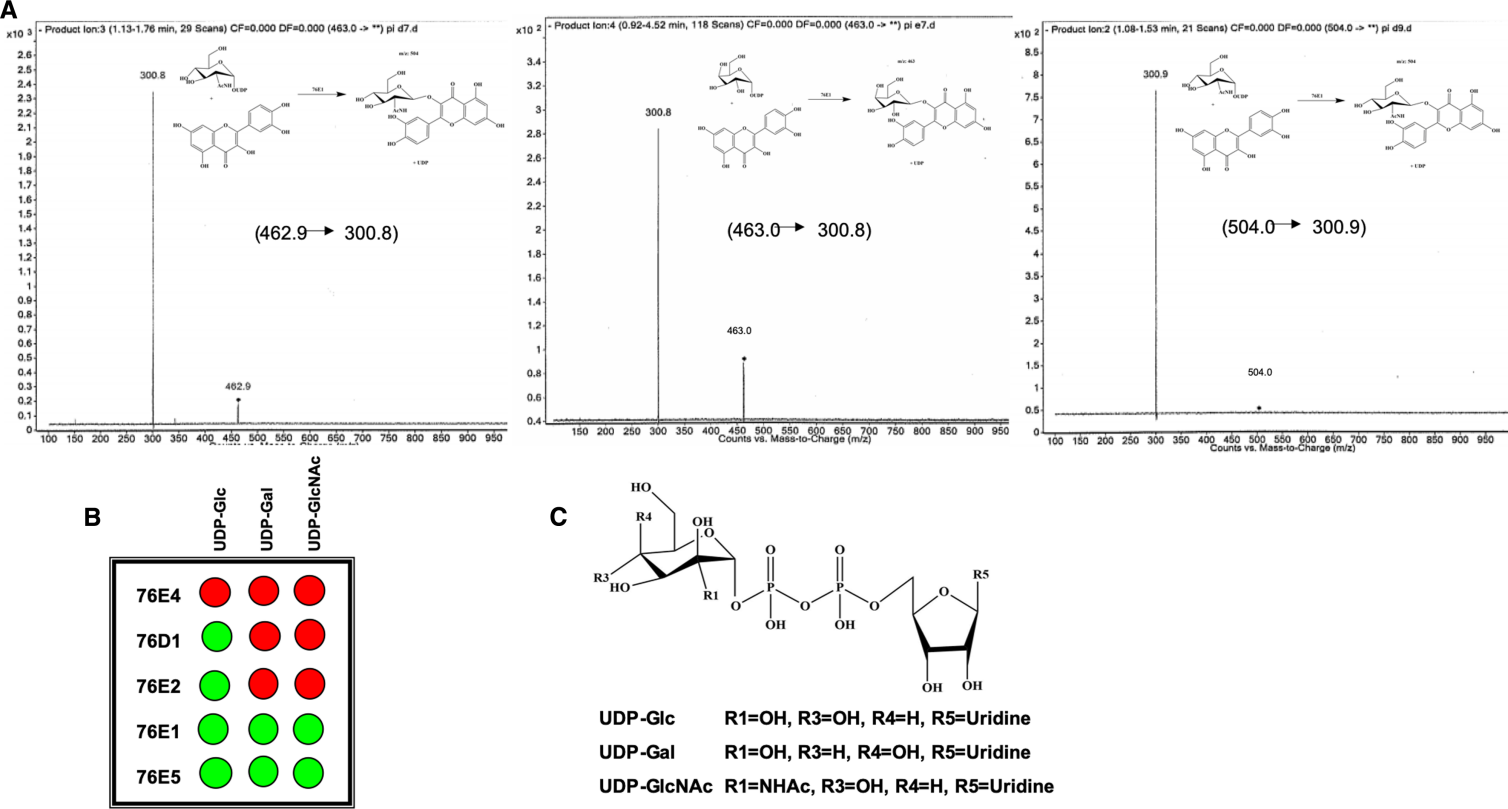
Product formation catalyzed by UGT 76E1. (**A**) MS/MS method to determine product formation using UDP-Glc, UDP-Gal, and UDP-GlcNAc with quercetin catalyzed by UGT 76E1; (**B**) GAR screen results showing summary of wildtype UGTs 76E4, 76D1, 76E2, 76E1, and 76E5 donor activities where green and red indicate positive and no activity, respectively; (**C**) structures of donor compounds used in this screening.

Samples were analyzed with Agilent 6400 triple quadruple mass spectrometer coupled with HPLC system using phenomenex-C18 column (50 × 4.6 mm, kinetex 5u, 100A). The solvents used were as follows: (A) HPLC grade water containing 0.1% formic acid and (B) acetonitrile containing 0.1% formic acid. For the MS scan, both positive and negative spectra were obtained and run at a low rate of 0.4 ml min^−1^ in isocratic mode (10% A and 90% B). The injection volume was 10 ml, detection wavelength at 260 nm, and column temperature of 20°C. Other details include start and end mass of 100 and 1000, respectively, scan time of 500 and cell accelerator voltage of 7. The MS/MS (product ion scan) had a low rate of 0.5 ml min^−1^ at gradient mode (70% A: 30% B for 1 min, 55% A: 45% B for 1.50 min, and lastly 70% A: 30% B for 2.50 min). The injection volume was maintained at 10 ml and only negative spectra were obtained here. The precursor ions were fragmented for confirmation within the range of *m*/*z* 100–1000. Scan segment details are as follows: scan time 500, fragmentor 135, collision energy 15, and cell accelerator voltage 7. All analyses were done in duplicates. The mass spectra (product ion scan) for wildtype and mutants are shown in Figures 1 and 4, respectively.

### Comparative homology modeling

**Figure 2.**
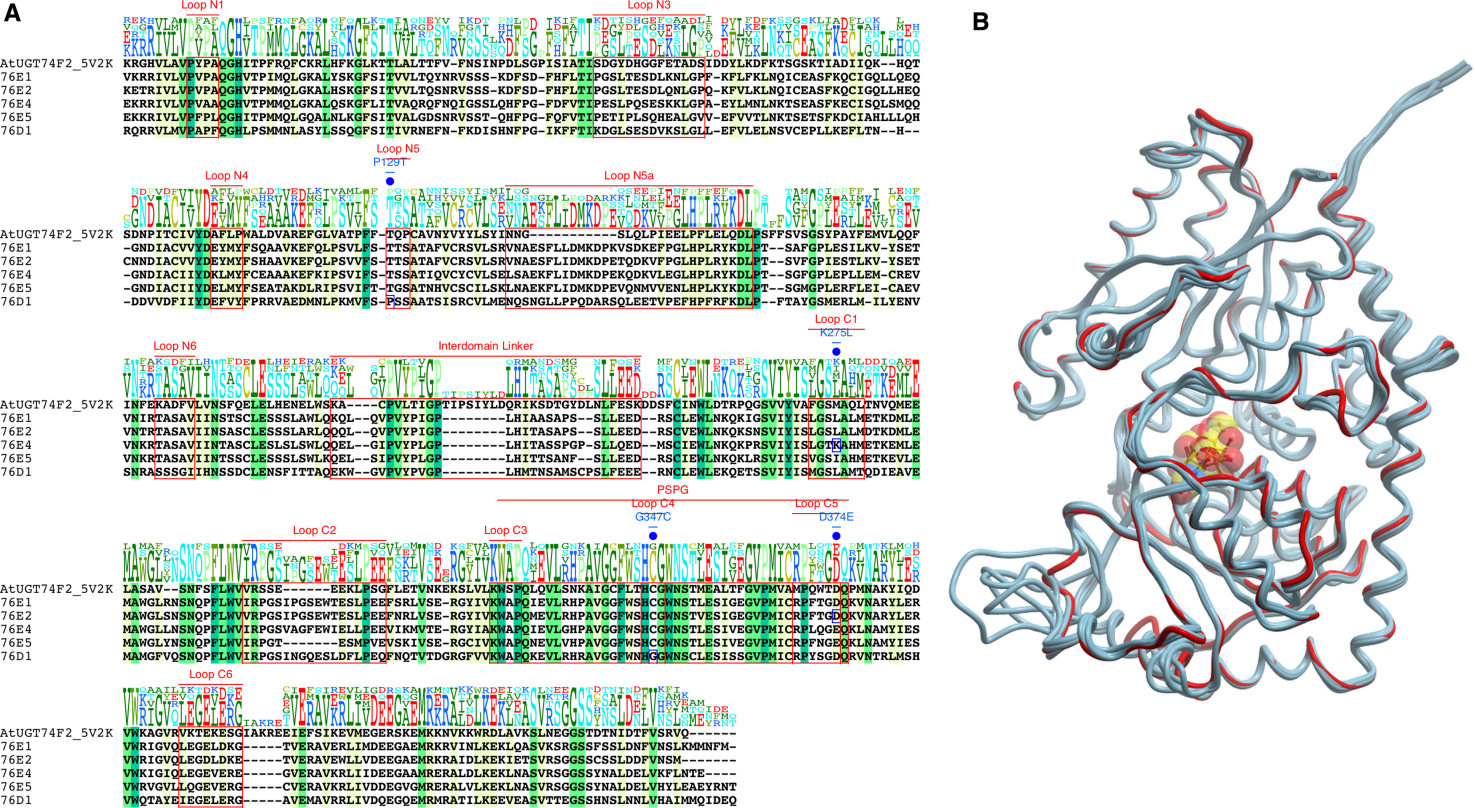
Sequence and structure comparison of group H *At*UGTs. (**A**) Multiple sequence alignment of *At*UGT74F2 with group H *At*UGTs reported in this study. Different regions are annotated above the sequences. The positions of mutation are marked in blue (**B**) Superimposition of 3D structures showing similarity between the models (cyan) and template *At*UGT74F2 (red). UDP-sugar is illustrated in CPK.

The sequences of the five UGTs, 76E1, 76E2, 76E4, 76E5, and 76D1, were obtained from UniProt (UniProt ID: Q9LTH3, Q9LTH2, Q9STE3, Q9STE6, and O48715, respectively). These sequences were used to search for structural homologs in the Protein Data Bank (PDB). Although there are a few templates available, the closest related 74F2 *A. thaliana* UGT (PDB ID 5V2K) was identified and the sequences were aligned using ClustalX [29]. The sequence identity between 74F2 and the five UGTs ranges between 30% and 33%. *At*UGT74F2 template structure has 444 residues, in which the N-terminal domain (residues 4–245) is connected to the C-terminal domain (residues 246–447) via an inter domain linker (232–250) [7]. The sequence alignment is illustrated in Figure 2.

The 3D models were generated based on the crystal structure of *At*UGT74F2 using the Modeller v 9.21 [30]. Loop refinement of the models was performed using the loop refinement tools in MolSoft ICM Pro 3.8-7c software (www.molsoft.com). The stereochemical parameters of the models were validated using the Ramachandran plot (Supplementary Figure S5) [31]. The modeled structures were further validated using ProSA II (Protein Structure Analysis) where *Z*-scores were calculated. *Z*-scores measure the compatibility between model sequence and structure and should be comparable to template's *Z*-score [32]. Pairwise structural superimposition of the models with the template *At*UGT74F2 was carried out using MolSoft ICM Pro 3.8-7c [33].

The spatial positions of sugar donor compounds UDP-Glc, UDP-Gal, and UDP-GlcNAc were built into the models using the ICM Pro 3.8-7c. The sugar donors were positioned in the models based on the orientation of UDP in the *At*UGT74F2 template. The side chains of the enzyme within 5 Å were energy minimized to relieve any steric clashes.

### Coevolution analysis

Evolutionary constraints on the residues in UGT 76D1 were estimated by residue coevolution analysis. From the FASTA sequence of the protein (UniProt ID: O48715), a multiple sequence alignment (MSA) was generated with a sequence search using hhblits from hhsuite3 [34] against UniRef30_2020_02 database. The alignment was further filtered using hhfilter to only include sequences with at least 75% coverage and maximum 90% identity. The residue coevolution matrix was calculated from the sequence alignment (23 104sequences) using ccmpred [35–37]. Pairwise coevolution scores were scaled, [38] analyzed, and plotted using in-house python scripts. All non-statistically significant (less than the average coevolution score) values were considered null.

### Molecular dynamics simulations

The parameters for the UDP-sugars were generated using Antechamber software implemented in the Amber Suite [39]. The geometry was optimized at the B3LYP/6-31G(d) level and the RESP charge fitting was done using electrostatic potential obtained at the HF/6-31G(d) level [40]. The parameters of the protein were defined using the ff14SB force field [41] and those of the ligands in GAFF2 [42]. The systems were set up via tleap, as implemented in the AmberTools18 suite. The complexes were solvated using TIP3P water model. The edge of the simulation box was set to 10 Å from the closest solute atom. Counterions were added to neutralize the net charge. Each system was minimized and equilibrated for 5 ns under NPT ensemble at 1 atm. The temperature was gradually increased to 300 K using a time step of 4 fs, rigid bonds, a cutoff of 9 Å and particle mesh Ewalds summations switched on for long-range electrostatics. Only the solvent and the ions were allowed to move during the equilibration. The backbone atoms of the protein and the ligands were constrained by a spring constant set at 1 kcal/mol/Å^2^. The production simulations were run in the NVT ensemble using a Langevin thermostat with a damping of 0.1/ps and hydrogen mass repartitioning scheme to achieve a time step of 4 fs. The production step was run for 50 ns without any constraints. A total of 20 replicates were run, with a total sampling time of 1 μs. Visualization of the simulation was carried out in VMD package [43] and figures made using ICM Pro suite [44] and Protein Imager [45]. The simulation protocol was identical for each system. A list of all simulations has been tabulated in Supplementary Table S2.

### Deep learning analysis

To further understand the molecular structures of different UGT variants acting on the three UDP-sugars, we employed a deep learning architecture, CVAE, to encode the high dimensional enzyme structures from the MD simulations into lower dimensional latent spaces. CVAE has been successfully applied to study the folding pathways of small proteins and structural clustering of biomolecules [46]. Each MD conformation of an enzyme variant was represented by a contact matrix using the corresponding key residues identified at the enzyme-substrate interface. The residues of 76E1 are 14–15, 109, 129–130, 266–269, 343–348, and 365–370; residues of 76E2 are 14–15, 110, 130–131, 266–271, 330, 344–349, 353, 368–369, and 372; residues of 76E4 are 14–15, 130–131, 267–271, 330, 345–350, 353, 367, 369, 370–371, and 373; residues of 76E5 are 12–15, 129–130, 265–269, 336, 338–341, 344, 358–361, and 364; residues of 76D1 are 13–15, 17, 125–126, 264–267, 342, 344–347, 350, 364–367, and 370. A pair of residues are defined as a contact if the distance between their Cα atoms is less than or equal to 8 Å. To ensure the contact matrices of the same size, we applied padding of 1 in both the *x*- and *y*-directions for the matrices of 76E1 and 76D1. The size of each matrix was, therefore, 22 × 22. We then (i) merged the contact matrices of different enzyme variants, (ii) randomly split the matrices into training and validation datasets using the 80/20 ratio, (iii) applied a CVAE to capture the important contact features, and (iv) projected them in the latent space for visualization. The encoder network of each CVAE consisted of three convolutional layers and a fully connected layer. We used a 3 × 3 convolution kernel and a stride of 1, 2, and 1 at the three convolutional layers, respectively. We trained each CVAE with RMSProp optimizer, using a learning rate of 0.001, until the training and validation loss converged. T-distributed stochastic neighbor embedding (t-SNE) was used to visualize the latent space in two dimensions [47].

## Results

We carried out *in vitro* characterization of five recombinant UGTs from *A. thaliana* group H, namely 76E1, 76E2, 76E4, 76E5, and 76D1, employing a MS-based method to examine their substrate specificities via monitoring the formation of reaction products qualitatively. MS has the benefit of directing a label free assay, permitting a speedy determination of enzyme substrate specificity without altering the reaction [48]. The main goal of this qualitative analysis is to identify the formation of intended glycosylated product against different substrates of interest catalyzed by the target UGTs. The presence of the glycosylated product indicates positive activity of a UGT against a substrate. Using the MS full scan analysis mode, a total ion current (TIC) plot is obtained which indicates all compounds present (in form of peaks molecular mass as well as signal intensity).

Furthermore, a MS/MS analysis using the selected ion monitoring (via the product ion mode) was used to confirm detection of the product. This is more precise as only selected compounds are detected and plotted. Without MS/MS confirmation, the possibility of a false signal exists in the first step (full scan analysis). Initial glycosylation activity was checked using protein lysate. UDP-Glc was set up as the donor sugar alongside with kaempferol and scopoletin as acceptor compounds. Once the glycosylation was confirmed, a sugar donor library containing three nucleotide sugars UDP-Glc, UDP-Gal, UDP-GlcNAc was set up for donor specificity screening.

### Donor specificity

Although UGTs are generally specific with donor sugar preference, activity with donors other than UDP-Glc is widely known [4]. Therefore, all five UGTs were screened through three donor sugars (Figure 1C) to establish their preferences. Mass spectra indicating usage of all three donor sugars in UGT 76E1 were shown in Figure 1A. The GAR screen [49] results are presented in Figure 1B. UDP-Glc was the most commonly used donor for the screened UGTs and is in agreement with previous research which highlighted its frequent use among plant UGTs as the favored donor compound [50]. UGTs 76E1, 76E2, 76D1, and 76E5 were able to use UDP-Glc as their substrate, consistent with our previous finding [28]. Unlike others, 76E4 showed no activity with any of the donor sugars screened.

Broad donor activity was observed for 76E1 and 76E5. Given that the structural flexibility of an enzyme's active site can affect its substrate recognition [51], the broad donor activity of both UGTs might due to their structural flexibility in the C-terminal domain. In addition, the structural differences of the donor sugars may affect the binding site. 76E1 and 76E5 also showed positive activity with UDP-Glc and UDP-Gal. Structurally, UDP-Gal differs slightly from UDP-Glc, mainly with the hydroxyl group at the C4 position orientating at a different direction. Sensitivity to this change in C4–OH's orientation by the binding site residues may influence an enzyme's specificity for this sugar donor. 76E2, 76D1, and 76E4 had no activity with UDP-Gal, which might be associated with their low tolerance for the orientation change of the OH group at the C4 position.

On the other hand, the active sites of 76E1 and 76E5 were able to accommodate the change. Specificity for UDP-Gal has been puzzling due to insufficient data to determine which plant UGT will accommodate the donor sugar. It has been suggested that a fine interplay of stereochemistry and conformation of donor sugars with UGT may likely be involved in UGT sugar specificity [13,52].

With an N-acetyl group on the C2 instead of an –OH, UDP-GlcNAc is larger in size than UDP-Glc. As the functional group is larger and more polar, it often makes substantial steric barrier compared with Glc [21]. Therefore, majority of UGTs are unable to accommodate UDP-GlcNAc. Our findings indicate that only 76E1 and 76E5 exhibited UDP-GlcNAc activity, suggesting that their active sites are large enough to accommodate the donor sugar's C2 positioned N-acetyl group. The mechanism dictating sugar donor as well as acceptor specificity and activity is multifaceted. Better understanding of the determinants of donor sugar specificity will become clearer as more crystal structures of plant UGTs of broader sugar recognition are solved. This should, in turn, advance and simplify structure prediction using comparative homology modeling.

### Sequence and structural analysis — comparative homology modeling

To rationalize structural differences resulting from donor specificity we constructed homology models. *At*UGT74F2 was chosen as a template to model group H UGTs as it belongs to the same family as the studied UGTs (Figure 2A). The template was exclusively selected over *Mt*UGT85H2 (with slightly higher sequence identity) due to the presence of substrate in its structure. Such a template permits the transfer of a prefabricated sugar-binding site and all associated conformational changes into the built model. The presence of an available binding site is, therefore, a prerequisite for successful docking of ligands [53].

Residues 6–448 (76E1), 7–443 (76E2), 6–443 (76E4), 6–442 (76E5), and 5–448 (76D1) were modeled based on *At*UGT74F2 template (residues 4–447). The sequence identity between *At*UGT74F2 and 76E1 76E2, 76E4, 76E5, and 76D1 ranges between 30 and 33% and the root mean-squared deviation between the template structure and models is between 0.4 and 0.8 Å (Figure 2B).

An assessment of the modeled protein structures is vital to highlight the overall quality and identify regions that may require further careful investigation. A Ramachandran plot can verify the details of the stereochemistry of structures, both experimentally solved and models. The plots for each of our models are shown in Supplementary Figure S5. Residues in the most favored regions are between 85% and 90% in all models, which confirms the overall good quality of the homology models. In addition to the Ramachandran plots, ProSA validated the overall model quality by a *Z*-score, which can then be compared with the scores of native proteins of similar size [32,33].

The *Z*-scores of the models fall within the range of the *Z*-scores of the existing crystal structures (Supplementary Figure S5), which further highlights the good quality of the homology models.

### Mutagenesis study

To rationalize substrate specificity among the five group H UGTs, MSA was carried out. An MSA, in conjunction with structural superimposition of the models, can highlight conserved structural positions, which participate in binding and substrate recognition [54]. Differences in conservation of key amino acids around the UDP-sugar binding site were investigated. Residues that interacted directly with the substrate were marked as potential key sites for investigation. In total, 14 amino acid side chains lie within 5 Å of the donor sugar binding site (Supplementary Table S3 and Supplementary Figure S6). These amino acids were thought to affect donor substrate recognition. Analyzing the differences allow structural rationalization and the design of novel mutants whose donor substrate recognition activity can be compared with the wildtype UGT activity. The loss or retention of original activity as well as acquisition of new activity may explain the role of a particular amino acid. In this study, the activity comparison was limited to mainly qualitative analysis.

**Figure 3.**
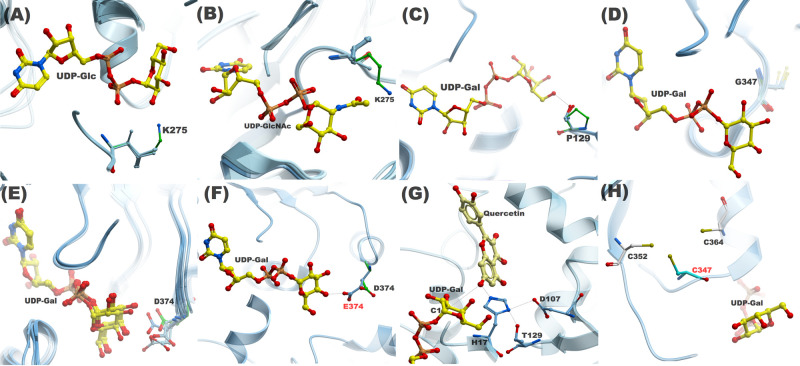
A structural comparison of WT and mutant residues investigated in this study. The proximity of K275 (green) side chain in C1 loop to (**A**) Glc C2–OH and (**B**) NAc group in 76E4; (**C**) P129 on N5 loop in 76D1 is unable to make hydrogen bond with the donor sugar due to the absence of the OH group; (**D**) Spatial position of G347 on C4 loop in 76D1; (**E**) Asp or Glu is present in C5 loop at equivalent position of D374 (76E2) in other group H *At*UGTs; (**F**) D374 side chain in 76E2 is unable to make hydrogen bond with the donor sugar. The side chain extended by one carbon atom to E374 enables the formation of a hydrogen bond; (**G**) The role of T129 in UDP-Gal recognition in 76D1 P129T mutant. T129 interacts with H17 and is involved in the formation of a catalytic triad, which is essential to glycosylation activity; (**H**) The proximity of C347 side chain to C352 and C364 side chains, where the Cys residues can potentially form a disulfide bond.

Our first set of MS experiments and GAR screen indicated that 76E1, 76E2, 76D1, and 76E5 were able to use UDP-Glc as a substrate. However, 76E4 did not display any activity with either of the donor sugars screened. The only notable difference between the residues that directly interacted with UDP-Glc is K275 in the C1 loop of 76E4 (Figure 3A). The equivalent residue is L273 in 76E1, L275 in 76E2, I273 in 76E5, and L270 in 76D1, respectively. They are all hydrophobic at this position. Structural comparisons indicated the orientation of the side chain of K275 points towards the C2–OH group in the glucose moiety. The lysine side chain could sterically impede the binding of donor sugar or could make direct interactions that prevent the substrate from leaving the binding site. Furthermore, this structural interference by the lysyl side chain also affects the binding of UDP-Gal and UDP-GlcNAc. We, therefore, decided to mutate 76E4 K275 to a leucine and study its donor sugar activity (Figure 3B).

While 76E1 and 76E5 displayed positive activity with UDP-Gal, 76E2, 76E4, and 76D1 did not show any donor sugar activity. We compared the alignment amongst the studied UGTs and identified P129 in the N5 loop of 76D1 (Figure 3C). P129 is replaced by a threonine (76E1—T134; 76E2—T136; 76E4—T135; 76E5—T134) at the equivalent position in all other UGTs. P129 is the first residue in the short N5 loop and we reasoned that the cyclic imino acid side chain of P129 might contribute to the rigidity of the N5 loop, and thereby preventing the accommodation of UDP-Gal and UDP-GlcNAc at the binding site. Additionally, a lack of –OH group in the side chain prevents this imino acid from making any direct interactions with the sugar moiety. A P129T mutant was subsequently made for 76D1 to test experimentally. Another striking difference was identified at position 347. A glycine residue is present in 76D1, while G347 is substituted by a cysteine in the other four UGTs (Figure 3D). This residue was mutated and the role of G347 on the donor sugar activity was studied. Glycine is known to enhance flexibility of secondary structures. Thus, its presence in the C4 loop might contribute towards destabilization of UDP-Sugar at the binding site.

Finally, to study 76E2 enzymatic activity, we carried out a subtle D374E mutation in the C5 loop (Figure 3E). This is a highly conserved position in the studied UGTs, which can accommodate either an aspartate or glutamate side chain. A structural comparison highlighted that the side chain of D374 is further away and does not make any direct interactions with the –OH groups of the sugar. Therefore, extending the side chain by a single carbon atom from aspartate to glutamate should bring the side chain closer and permit direct interaction with the –OH groups of the sugar (Figure 3F).

**Figure 4.**
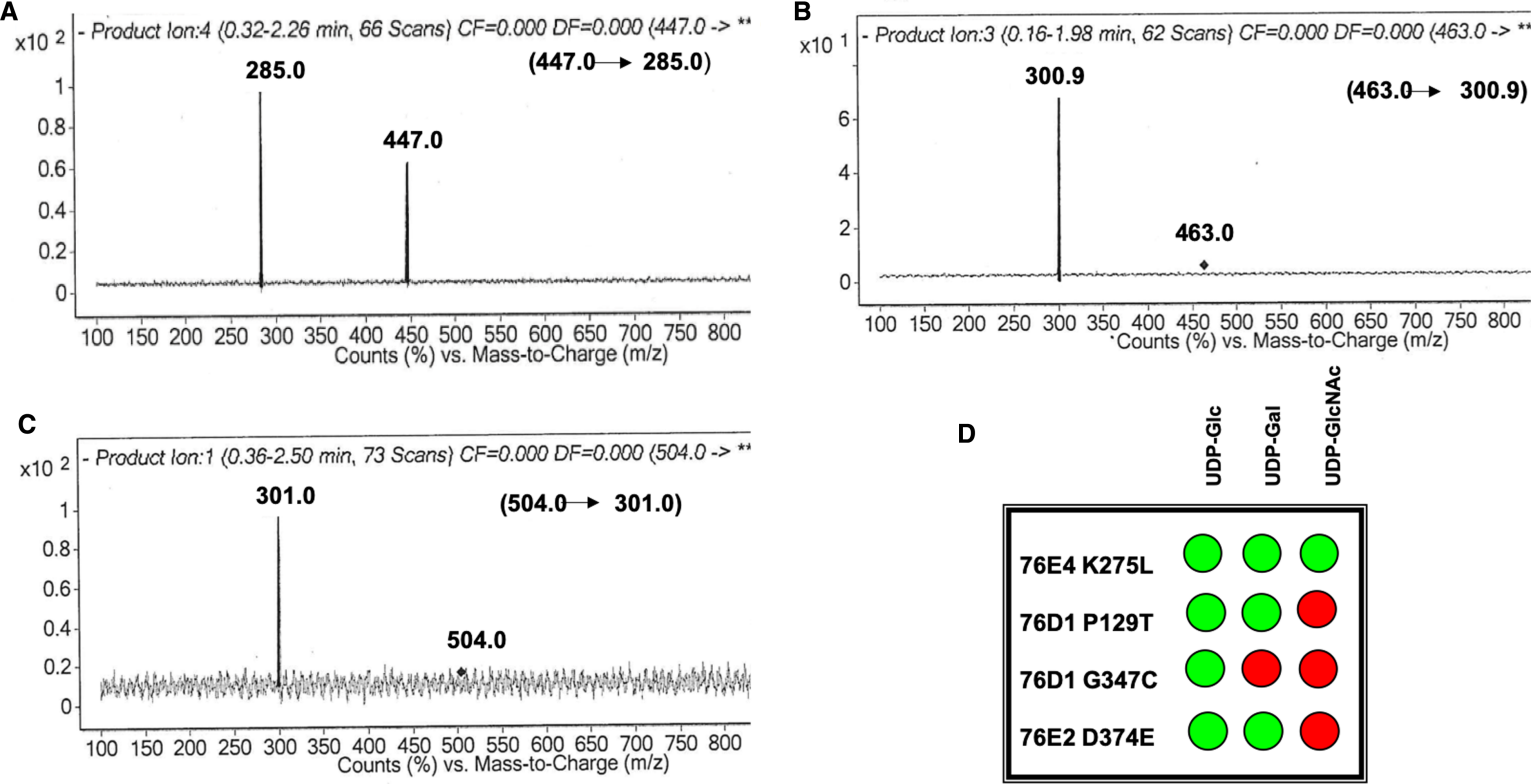
Confirmation of product formation. Mass spectra—product ion scan confirming glycosylation in mutant 76E4 K275L with (**A**) UDP-Glc, (**B**) UDP-Gal and (**C**) UDP-GlcNAc donor sugars; (**D**) GAR screen results showing summary of mutant UGTs 76E4 K275L, 76D1 P129T, 76D1 G347C, and 76E2 D374E donor activities where green and red indicate positive and no activity, respectively.

Mutant UGTs 76D1 P129T, 76E4 K275L, 76E2 D374E, and 76D1 G347C were designed and produced. Subsequently, a donor activity screening was carried out using the same sugar donors, UDP-Glc, UDP-Gal, and UDP-GlcNAc, with acceptor substrates quercetin and kaempferol. Liquid chromatography-mass spectrometry/mass spectrometry (LC–MS/MS) was used to observe reaction products, hence, to confirm sugar addition. The findings are summarized in Figure 4D. 76E4 K275L mutant was able to recover donor activity for all three sugars. The mass spectra are indicated in Figure 4A–C. 76D1 P129T and 76E2 D374E displayed positive activity with UDP-Glc and UDP-Gal but no activity for UDP-GlcNAc. 76D1 G347C was only positive to UDP-Glc.

### Molecular dynamics simulations and deep learning

To further elucidate the dynamic structures of UGT residues in sugar donor activity, we carried out MD simulations of the complexes in explicit solvent. Supplementary Table S2 summarizes the simulations run for each system. In total, we sampled 23 μs of MD simulations.

To gain further insights into the relationship between molecular structure and substrate specificity of different UGT variants, we applied CVAEs to encode the high dimensional enzyme structures from the MD simulations into lower dimensional latent spaces and visualized the contact features embedded in the latent spaces by t-SNE [47].

**Figure 5.**
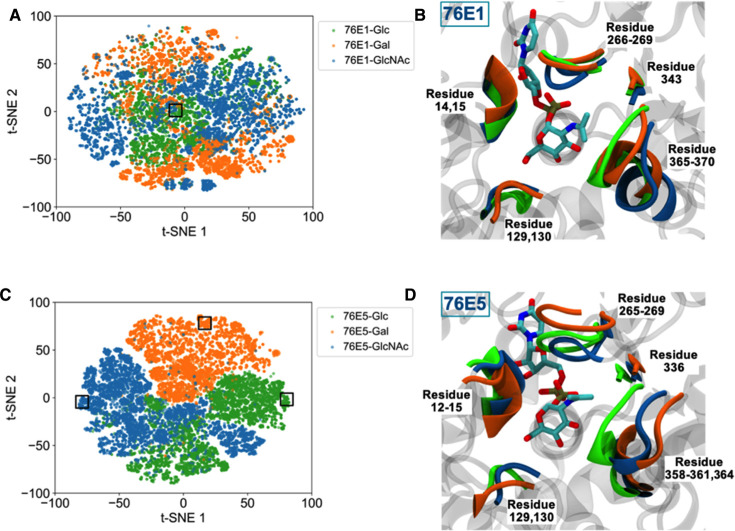
Structural profiles of UGTs 76E1 and 76E5 against the three sugar substrates. The 2D t-SNE plots illustrate the latent spaces of (**A**) 76E1 and (**C**) 76E5. The catalytic residues are highlighted in the cartoon representation in (**B**) and (**D**) taken from the representative 3D structures outlined in boxes in the t-SNE plots. UDP-GlcNAc is shown in stick representation. The enzyme structures are color coded, where UGT-(UDP-Glc) is in green, UGT-(UDP-Gal) in orange, and UGT-(UDP-GlcNAc) in blue.

A detailed description of how we prepared the input datasets and performed the training can be found in Methods. Based on the GAR screening results, UGTs 76E1 and 76E5 exhibit positive activity for all three sugar substrates among the five wildtype UGTs. Nevertheless, the two enzymes illustrate distinct structural profiles against the three substrates (Figure 5).

The contact features of the UGT 76E1 bound by the three sugar substrates are indistinguishable in the latent space (Figure 5A). There is no distinct cluster corresponding to substrate specificity. From the representative 3D structures, the catalytic residues arrange similarly despite different substrates at the binding site (Figure 5B). On the other hand, the residues of UGT 76E5 depict different structural arrangement upon different substrate bindings. There is clear separation between the clusters in the latent space (Figure 5C) and distinct structural displacement of the catalytic residues in the corresponding 3D structures (Figure 5D).

**Figure 6.**
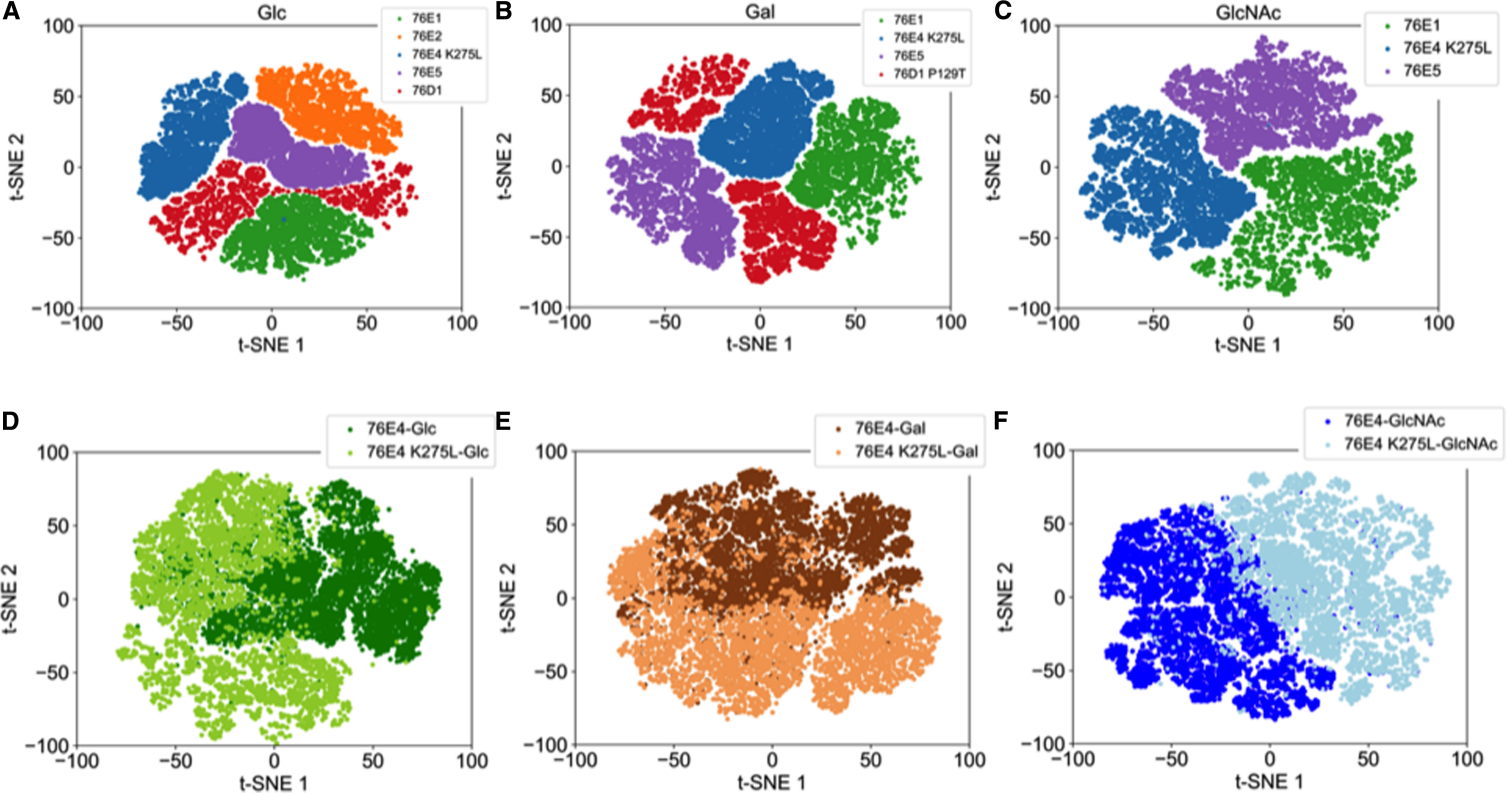
Structural profiles of UGT variants based on substrate specificity. The 2D t-SNE plots illustrate the latent spaces of the enzymes exhibiting positive activity for (**A**) UDP-Glc, (**B**) UDP-Gal, and (**C**) UDP-GlcNAc. The 2D t-SNE plots illustrate the latent spaces of the wildtype UGT 76E4 and the mutant 76E4 K275L against (**D**) UDP-Glc, (**E**) UDP-Gal, and (**F**) UDP-GlcNAc.

In addition, different UGTs with the same substrate activity illustrate different structural profiles. For example, all UGTs 76E1, 76E2, 76E4 K275, 76E5, and 76D1 show positive activity toward UDP-Glc, and they have distinct contact features embedded in the latent space (Figure 6A). Similarly, all UGTs show distinct latent space profiles for UDP-Gal and UDP-GlcNAc (Figure 6B,C). The wildtype UGT 76E4 shows negative activity against the three sugar substrates but introducing a point mutation from lysine to leucine at residue 275 restores enzyme activity toward the three substrates. It is known that a single point mutation can result in large conformational changes in protein structure [55,56]. However, in UGT 76E4 the point mutation does not affect global protein structure but rather local structure as shown in the contact features of the critical residues in the latent spaces (Figure 6D–F).

## Discussion

Uridine diphosphate GTs have shown promising potential as catalysts in the synthesis of medically relevant glycosides [5]. However, incomplete understanding at the molecular level due to insufficient biochemical and structural information has hindered potential applications of most of these UGTs. More biochemical data can improve our understanding of their substrate preferences. Additionally, due to the wide range of potential substrates that needs to be screened, biochemical characterization of the substrate specificity is also demanding [1].

A more targeted and faster means to understand substrate specificity of UGTs is to study their interactions with the substrate from 3D structures [17]. Since only a limited number of solved structures of plant UGTs are available, homology modeling using solved crystal structures as templates suggests an alternative method [4,17,57]. Recently, extensive development has been made in structural studies of plant UGTs. A thorough look at plant UGTs capable of recognizing diverse donor sugars of UDP, GDP, and dTDP base such as UGT89C1 [58] could help into understanding the difference in the substrate flexibility. This UGT's crystal structure in complex with UDP-rhamnose and acceptor quercetin has been recently solved [16], which improves our understanding of the mechanism of glycosylation and UGT engineering to aid the production of bioactive glycosides. In plant UGTs, the C-terminal domains are highly similar leading to their recognition of same or similar donors. Few other regions in the N-terminal have been shown to also participate in donor sugar recognition. Manipulation of these regions influences activity and varies donor sugar specificity in UGTs [6].

Wildtype 76E4 displayed no glycosylation activity in our donor screening with UDP-Glc, UDP-Gal, and UDP-GlcNAc (Figure 1B**)**. However, mutant 76E4 K275L recognized all donor sugars (Figure 4A). The structural difference observed in the C1 loop (lysine in place of leucine/isoleucine) predicted this mutation. The important role of C1 loop in substrate recognition has been previously reported [1]. In UGT71G1, mutation of M286L in the C1 loop improved substrate recognition and increased activity with UDP-Gal and UDP-glucuronic acid [27].

The 76E4 K275 lysyl side chain is positioned adjacent to the C2–OH group and sterically hinders the position of sugars at the binding site (Figure 3A,B). Additionally, L275 is positioned adjacent to T274, which interacts with the UDP-phosphate group. Its spatial proximity to the phosphate group and the C1 reaction center of the donor sugars may contribute to the improved donor specificity (Supplementary Figure S4C). As identified in our UGTs as well as the 10 solved plant UGTs structures, the conserved serine/threonine in the C1 loop typically has a hydrophobic residue adjacent to it (Supplementary Figure S4B). The conserved serine/threonine in this loop generally forms hydrogen bonds with the UDP-phosphate group in plant UGTs [7].

For glycosylation to occur, a catalytic triad is established between H17 and D107 of UGT and 3-OH of quercetin, which must also be near the C1 atom of donor sugar. The hydrogen bond between T129 and H17 may aid to confer UDP-Gal specificity to the mutant UGT 76D1 P129T (Figure 3G). Structurally, the cyclic imino acid proline is restricted in making interactions like other amino acids and therefore resides in very tight turns in protein structures. On the other hand, threonine is polar, fairly reactive and able to form hydrogen bonds [59]. T129 may have increased flexibility to the conformation conferred on the protein by proline structure. This allows the protein to adopt conformations that helped in UDP-Gal recognition. T129 forms hydrogen bonds with other than C6–OH of donor sugar moiety, which may have helped improve its activity such as shown in Figure 3C.

76E2 D374E mutant displayed a UDP-Gal activity that was absent from the wildtype 76E2. Recognition of substrates like kaempferol was observed in the mutant UGT. Residue 374 is present on the C5 loop, which is towards the end of the PSPG motif (Supplementary Figure S3). 76E2 wildtype, however, only recognized UDP-Glc. Aspartic acid is very similar to glutamic acid as both are negatively charged, polar amino acids. However, glutamic acid has a longer side chain than aspartic acid by one carbon atom. Aspartic acid's shorter side chain confers slight rigidity within protein structures [59]. Similar to observations in a previous study [59,60], the additional methylene group of glutamic acid lowers the solubility of the side chain, which strengthens the charge-dipole interactions between the side chain and C4–OH. The longer side chain of glutamic acid assisted the mutant 76E2 D374E to achieve hydrogen bonding to UDP-Gal by moving closer to the C4–OH (Figure 3F). Molecular interactions explain that D374 in wildtype 76E2 did not interact with the C4–OH of UDP-Gal. However, E374 in mutant 76E2 D374E formed a hydrogen bond with C4–OH of UDP-Gal (Figure 3E,F). The C4–OH interaction is believed to be central to UDP-Gal activity, which may be why the wildtype did not recognize UDP-Gal.

76D1 G347C did not show any activity to UDP-donor sugars besides UDP-Glc. The structural role of cysteine at this position is unclear and did not confirm our hypothesis on the importance of the C4 loop cysteine on donor sugar recognition. There are two plausible explanations. First, there are two other cysteine residues near C347 at positions 352 and 364 (Figure 3H). C347 can potentially form a disulfide bond with either of C352 or C364, depending upon the spatial position of its side chain. A potential disulfide bond will enhance the rigidity of the PSPG region (increase stability) and ultimately decrease varied substrate recognition. Intra-domain interactions such as disulfide bridges can confer stability of secondary and tertiary structure of a protein, and they may be important for activity and specificity [1]. The location of a disulfide bridge within a structure may influence its role in the stabilizing or folding of the protein. They help to stabilize the native conformation and maintain protein integrity, making them less susceptible to denaturation and degradation [61]. In *Mt*UGT85H2, the stability of the PSPG motif region is said to be likely increased by the presence of a disulfide bridge between C349 and C366 [11]. This may be one explanation why there was no significant improvement in donor recognition of 76D1 G347C. Second, the presence of a pair of cysteine residues in the PSPG motif does not necessarily lead to the formation of disulfide bond as seen in *Vv*GT1 where PSPG motif residues C351 and C368 are far apart (∼3.4 Å) and do not form disulfide bridges [13]. To further understand both scenarios, we carried out coevolution analysis on residue G347 (Supplementary Figure S7). Statistically significant scores were observed for the residue-pairs G347-C352 and G347-C364. This suggests that the residue pairs have an evolutionary relationship and mutation on one of the sites may require a compensatory change in the other. G347 exhibits the strongest coevolution signal and its perturbation may require a compensatory change at position 352 and 364. In case of 76D1 G347C mutant, we have assumed this behavior and run simulations with no disulfide bond formed between G347C and neighboring cysteine residues.

Molecular simulations and deep learning analysis were performed to study the dynamic interactions between molecular structure and sugar donor specificity of group H UGTs. Even though 76E1 and 76E5 displayed positive activity for all sugar substrates, they exhibited distinct structural profiles. A comparison of differences between substrate specificities in 76E1 was limited since the binding site residues arranged in a similar manner for all three donor sugars. However, this was not the case in 76E5, where distinct substrate clusters were identified. Furthermore, different UGTs with the same substrate activity illustrated different structural profiles. This highlights the complex interplay between substrate recognition with subtle stereochemical differences and the importance of local structural changes in these enzymes.

## Conclusions

Enzyme engineering via structure-guided mutation at the active site of UGTs has been shown to manipulate enzyme activity and substrate specificity, generating new UGTs with enhanced function. Besides the benefit of synthesizing bioactives, structure-based enzyme engineering also helps plant metabolic engineering towards improving the production and quality of crop plants [26]. While selecting amino acids for mutation, any structural, biochemical, or protein sequence information may be valuable. A typical approach is to concentrate on catalytic region. Amino acids that modify substrate specificity are usually non-conserved residues which often near catalytic residues or active sites. Another way is to identify conserved sequence motifs [12,14,15].

Obtaining crystal structure can be time-consuming and challenging. In the absence of experimental crystal structures, we employed advanced molecular modeling, simulations, and deep learning analysis in conjunction with biochemical characterization to design a workflow that can be used to study plant UGTs. Based on our rational structural manipulation and analysis, we identified some key amino acids, which improved substrate recognition. Mutant UGTs such as 76E4 K275L, 76D1 P129T, and 76E2 D374E showed improved donor recognition than their wildtype UGTs. The design of these UGTs with broader substrate specificity may play important role in their biotechnological and industrial applications. These findings are believed to serve as basis for further studies of plant UGTs and thereby advancing UGT enzyme engineering.

## Competing Interest

The authors declare that there are no competing interests associated with the manuscript.

## Funding

AA was funded by the Federal Scholarship Board/Presidential Special Scholarship Scheme for Innovation and Development (PRESSID), Nigeria; SH and YC would like to acknowledge funding from Sichuan Science and Technology Program grant 2018HH0129.

## Open Access

Open access for this article was enabled by the participation of University College London in and all-inclusive *Read & Publish* pilot with Portland Press and the Biochemical Society under transformative publisher agreements.

## Author Contributions

S.H. designed the research; A.A. performed the wet-lab experiments, built the models; X.L. and Y.C. analyzed the simulation data; S.H.C. and D.B. performed the deep-learning analysis; S.C.D. and A.P. conducted the coevolution analysis; all authors contributed to the writing of the manuscript.

## Notice

This manuscript has been authored by UT-Battelle, LLC under Contract No. DE-AC05-00OR22725 with the U.S. Department of Energy. The United States Government retains and the publisher, by accepting the article for publication, acknowledges that the United States Government retains a non-exclusive, paid-up, irrevocable, worldwide license to publish or reproduce the published form of this manuscript, or allow others to do so, for United States Government purposes. The Department of Energy will provide public access to these results of federally sponsored research in accordance with the DOE Public Access Plan (http://energy.gov/downloads/doe-public-accessplan).

## Abbreviations

CVAE: convolutional variational autoencoder
MD: molecular dynamics
MS: mass spectrometry
MSA: multiple sequence alignment
t-SNE: by t-distributed stochastic neighbor embedding
UDP: uridine diphosphate
UDP-Gal: UDP-galactose
UDP-Glc: UDP-glucose
UDP-GlcNAc: UDP-N-acetylglucosamine
UGT: UDP-dependent glycosyltransferases;
