# Supplementary material for: Structure-based enzyme engineering improves donor-substrate recognition of Arabidopsis thaliana glycosyltransferases
Source: Biochem J. 2020 Aug 7;477(15):2791–805. doi: 10.1042/BCJ20200477 (PMC7419078; doi:10.1042/BCJ20200477)
Supplement: Supplementary Figures S1-S7 and Tables S1-S3 [file BCJ-477-2791-s1.pdf]

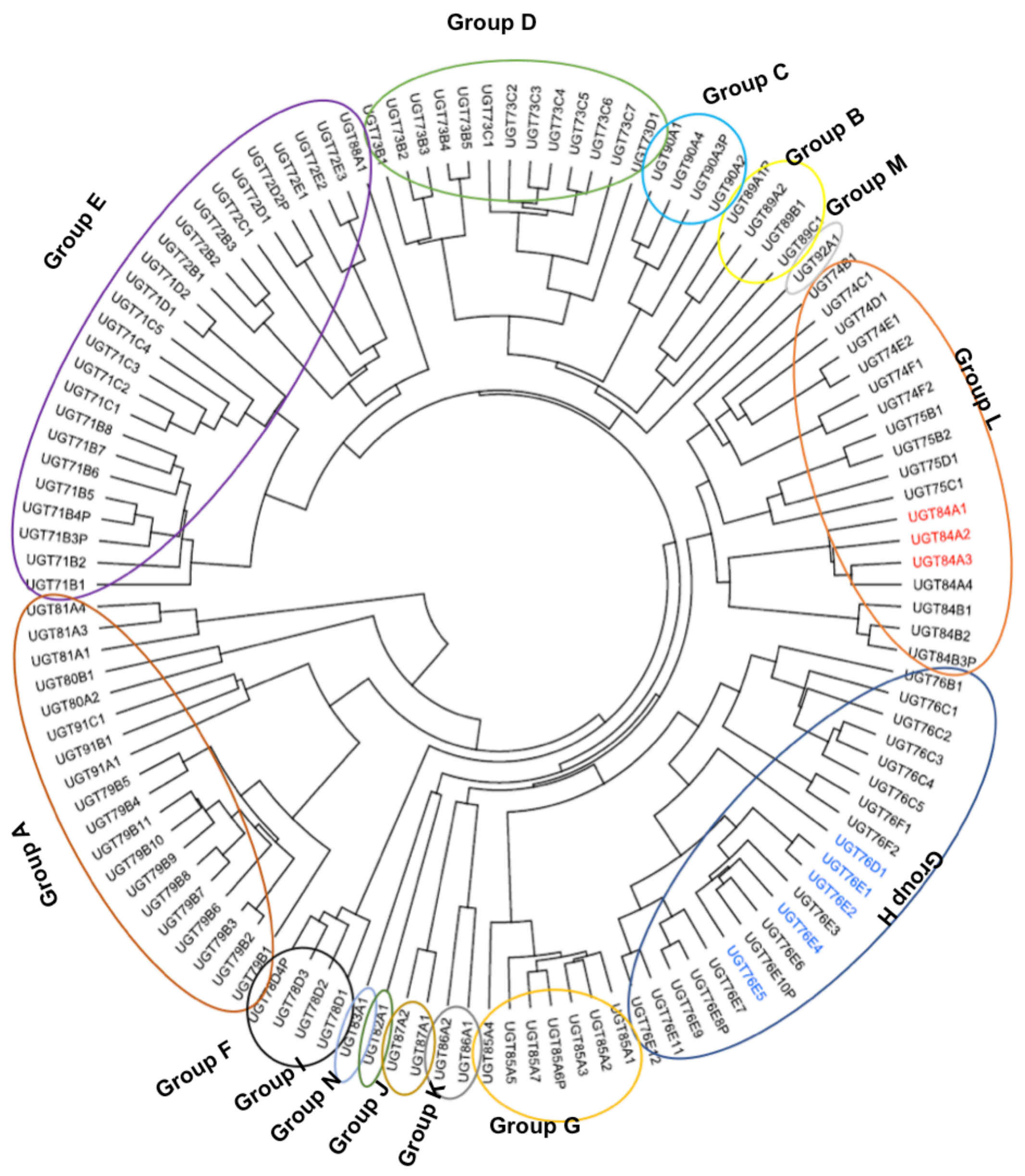

Figure S1: Phylogenetic tree showing 122 Arabidopsis thaliana UGT genes grouped into A-N (circled). Eight UGTs from groups H are printed in blue.

Plant UGT	PDB code	In complex with	Reference
***At*UGT74F2**	5V2K<br>5U6M<br>5U6N<br>5U6S<br>5V2J	(T15A) with UDP and 2-bromobenzoic acid<br>UDP and salicylic acid<br>(T15S) with UDP and salicylic acid<br>UDP and 2-bromobenzoic acid<br>(T15S) with UDP and 2-bromobenzoic acid	George-Thompson et al. (2017)
***At*UGT72B1**	2VCE<br>2VCH<br>2VG8	UDP<br>UDP<br>UDP-2-fluoro-glucose and TCP	Brazier-Hicks et al. (2007)
***Mt*UGT78G1**	3HBF<br>3HBJ	UDP and myricetin<br>UDP	Modolo et al. (2007)
***Mt*UGT71G1**	2ACV<br>2ACW	UDP<br>UDP-glucose	Shao et al. (2005)
***Mt*UGT85H2**	2PQ6	None	Li et al. (2007)
***Ct*UGT78K6**	3WC4<br>4REL<br>4REM<br>4REN<br>4WHM	None<br>Kaempferol<br>Delphinidin<br>Petunidin<br>UDP	Hiromoto et al. (2013), (2015)
***Vv*GT1**	2C1X<br>2C1Z<br>2C9Z	UDP<br>UDP-2-fluoro-glucose and kaempferol<br>UDP and quercetin	Offen et al. (2006)
***Os*79**	5TMB<br>5TMD<br>5TME<br>6BK0<br>6BK1<br>6BK2	UDP<br>U2F and trichothecene<br>UDP<br>Q202A with UDP<br>T291V with UDP<br>H122A/L123A with UDP	Wetterhorn et al. (2016), (2017)
***P*tigS**	5NLM	Magnesium ion and 3-sulfooxy-1H-indole	Hsu et al. (2018)
***At*UGT89C1**	6IJ7<br>6IJ9<br>6IJA<br>6IJD	None<br>UDP<br>UDP-rhamnose<br>Quercetin	Zong et al., (2019)

Table S1: Crystal structures of plant UGTs available in the protein data bank.

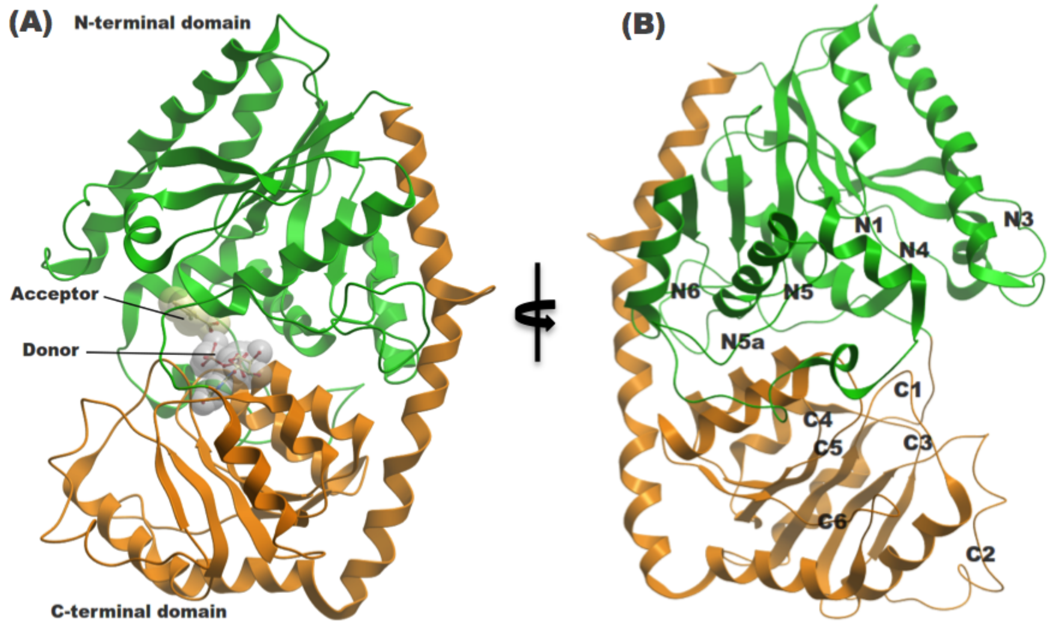

Figure S2: (A) Conserved GT-B fold of a representative of ten currently solved plant UGT crystal structures; The N- and C-terminal domains are (shown in green and orange respectively) with acceptor and donor substrates bound; (B) Loop regions of the N- and C-terminal domains in AtUGT74F2. These loop regions offer vital interactions with both acceptor and donor substrates in plant UGTs.

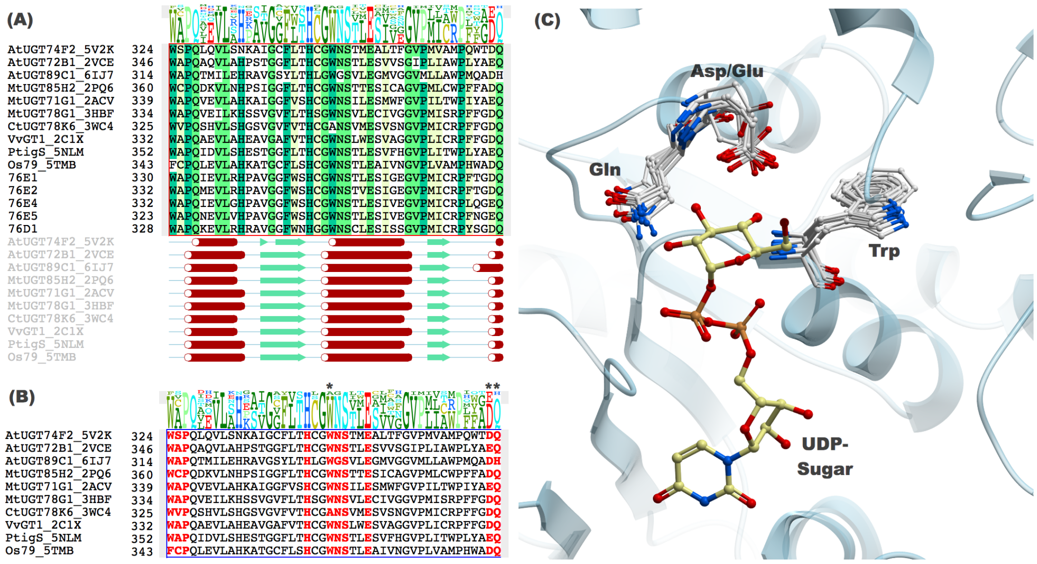

Figure S3: (A) Sequence alignment of the Plant Secondary Product Glycosyltransferase (PSPG) box, a characteristic structural feature of plant UGTs, from all ten crystal structures of plant UGTs solved and the five group H ***At***UGTs reported in this study; (B) Conservation of key amino acid residues in the PSPG box motif in the crystal structures that interact with the sugar; (C) Structural superimposition of conserved residues (Tryptophan, Aspartic acid/Glutamic acid and Glutamine) from the ten plant crystal structures which interact with the sugar part of the UDP- donor sugar.

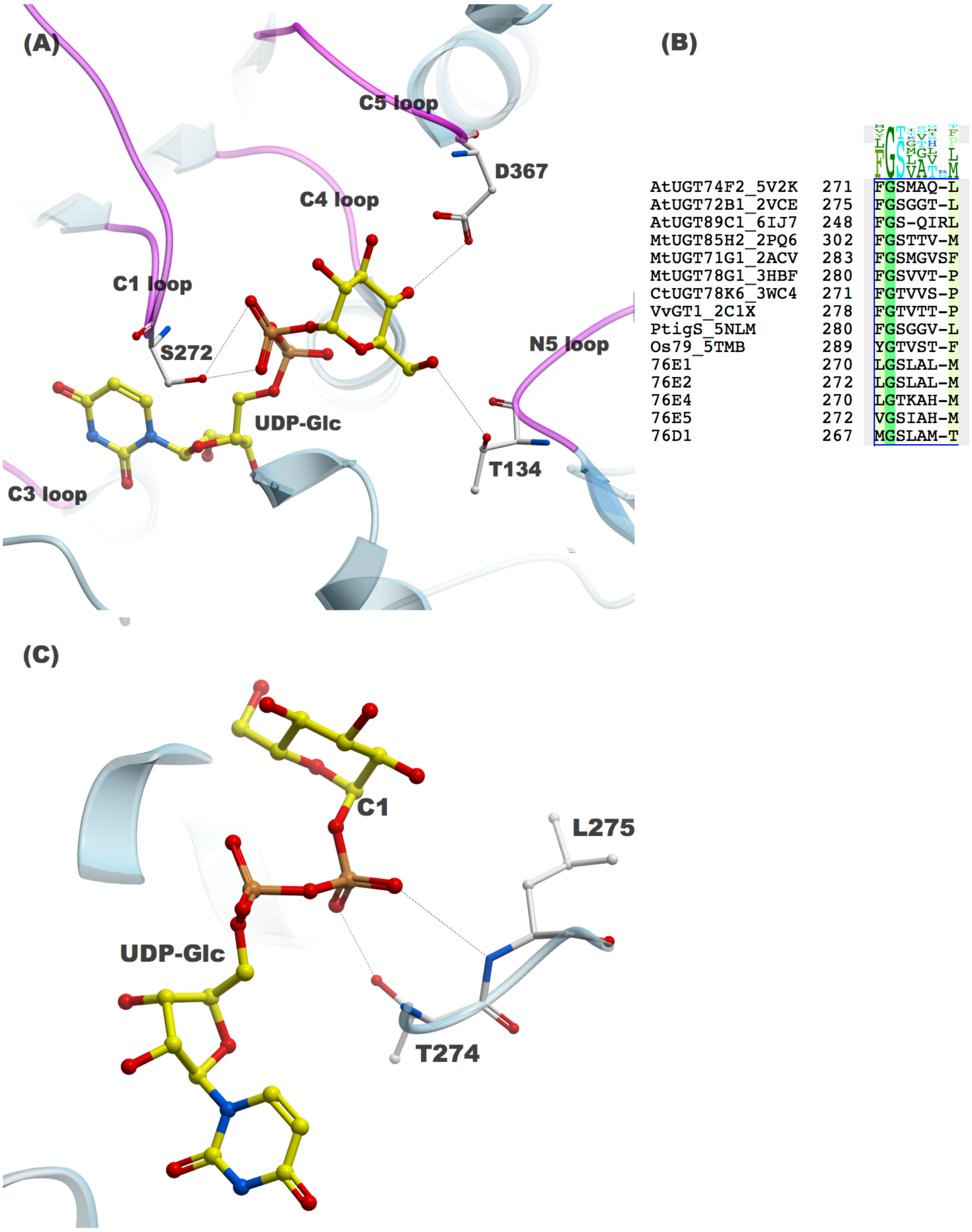

Figure S4: (A) Residues in loops from both C-terminal and N-terminal domain surround the UDP-sugar binding site and contribute to direct structural interactions; (B) Multiple sequence alignment highlighting C1 loop residues potentially involved in substrate recognition; (C) The spatial position of L275 in ***At***UGT76E4 K275L mutant, highlighting the proximity to the reaction centre of UDP-Glucose, which may have helped improved donor specificity.

System	Donor Sugar	Cα RMSD (Å)
76E1	Glc	4.01
	Gal	4.36
	GlcNAc	4.69
76E2	Glc	3.89
	Gal	4.59
	GlcNAc	3.87
76E2 D374E	Gal	4.52
	GlcNAc	4.13
76E4	Glc	4.93
	Gal	4.82
	GlcNAc	4.35
76E4 K275L	Glc	4.72
	Gal	4.58
	GlcNAc	4.22
76E5	Glc	4.23
	Gal	3.72
	GlcNAc	3.89
76D1	Glc	4.98
	Gal	3.89
	GlcNAc	4.28
76D1 P129T	Glc	4.27
	Gal	4.20
	GlcNAc	4.42

Table S2: Summary of simulations. All simulations were of 1µs each (see methods for details). RMSD of Cα were calculated from their positions in the starting structure and averaged over the total number of simulations.

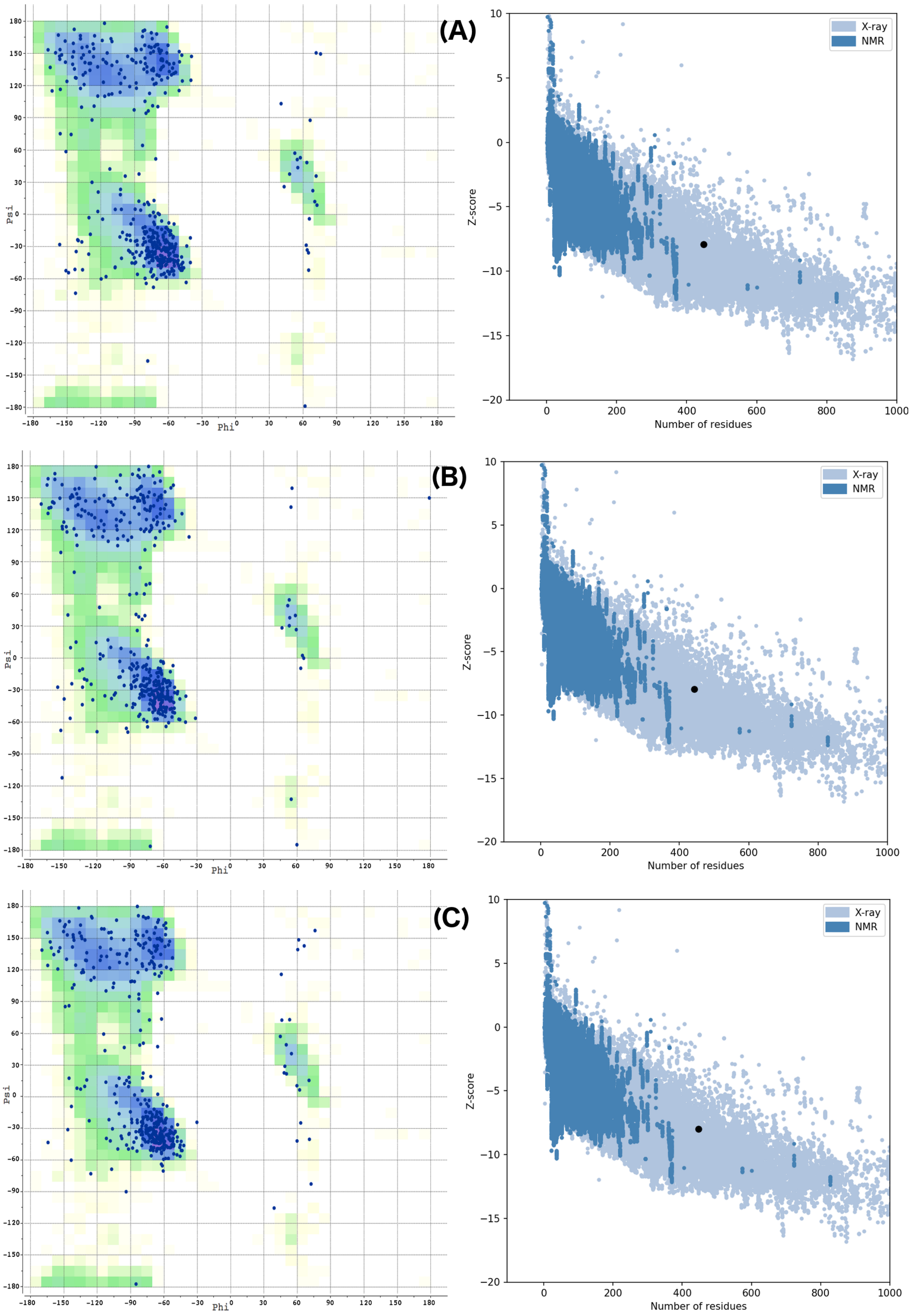

Figure S5: Stereochemical checks of the models. Ramachandran plots and PROSA analysis for models (A) 76E1, (B) 76E2, (C) 76E4. The black dot in each PROSA plot represents the Z-score. The Z-score is -7.91, -7.96 and -7.99 respectively.

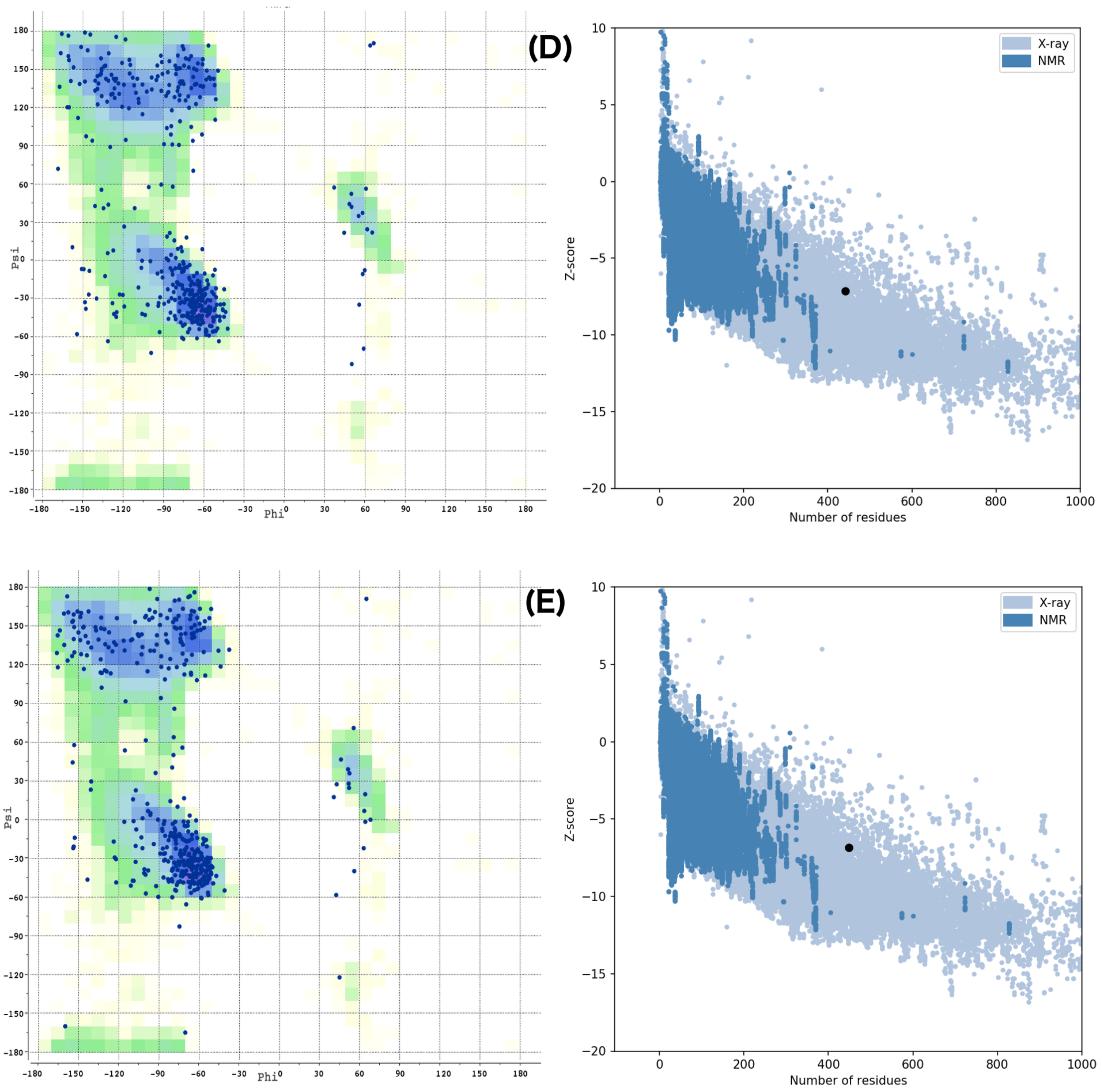

Figure S5 (contd): Stereochemical checks of the models. Ramachandran plots and PROSA analysis for models (D) 76E5 and (E) 76D1. The black dot in each PROSA plot represents the Z-score. The Z-score is -7.13 and -6.92 respectively.

*At*UGT	Residues within 5Å of sugar
76E1	H20, Y114, T134, L273,G371, D372, Q373, N376, H348, C349, G350, S351, S352, S353
76E2	K275, Y116,H21, T136, G373, D374, Q375, N378, H350, C351, G352, W353, N354, S355
76E4	K275, L115, H20, T135, G373, F374, H350, C351, G352, W353, N354, S355, Q375, N378
76E5	I273, L115, H20, T143, G364, E365, Q366, N369, H341, C342, G343, W344, N345, S346
76D1	L270, F109, H19, P129, G369, D370, Q371, N374, H346, G347, G348, W349, N350, S351

Table S3: Residues that lie within 5 Å of donor sugar

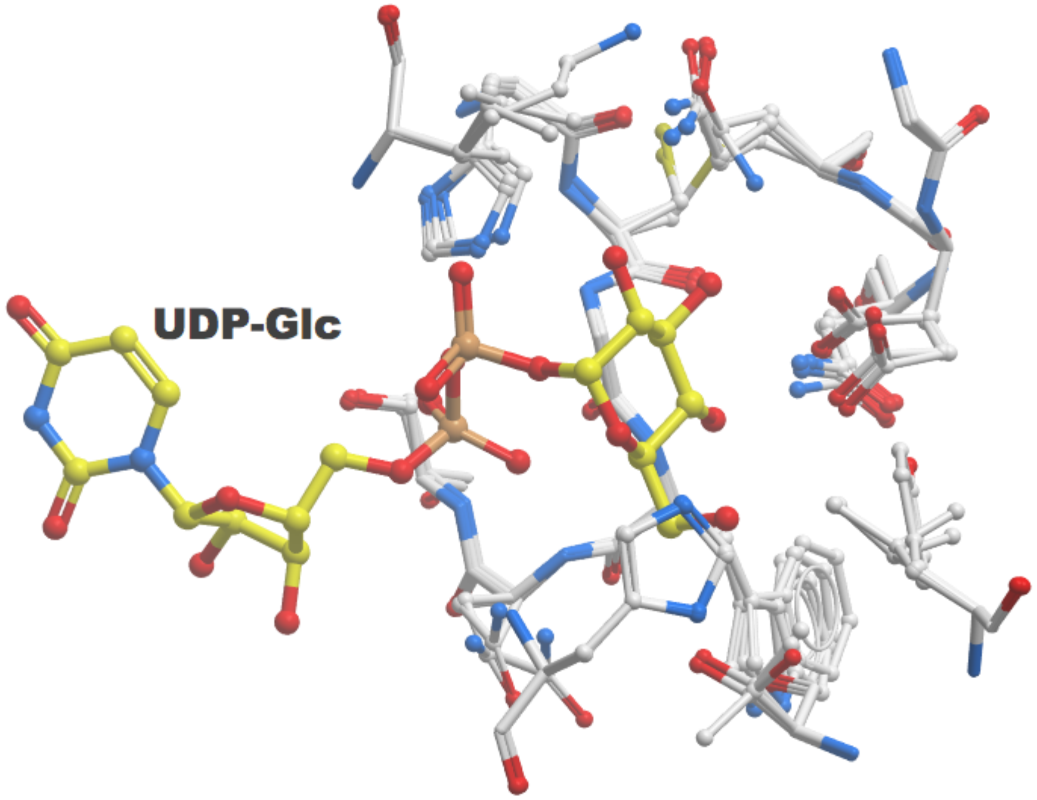

Figure S6: Side chains of 14 residues lie within 5Å of the donor sugar. All 5 ***At***UGTs have been superimposed on 76E1-UDP-Glc complex.

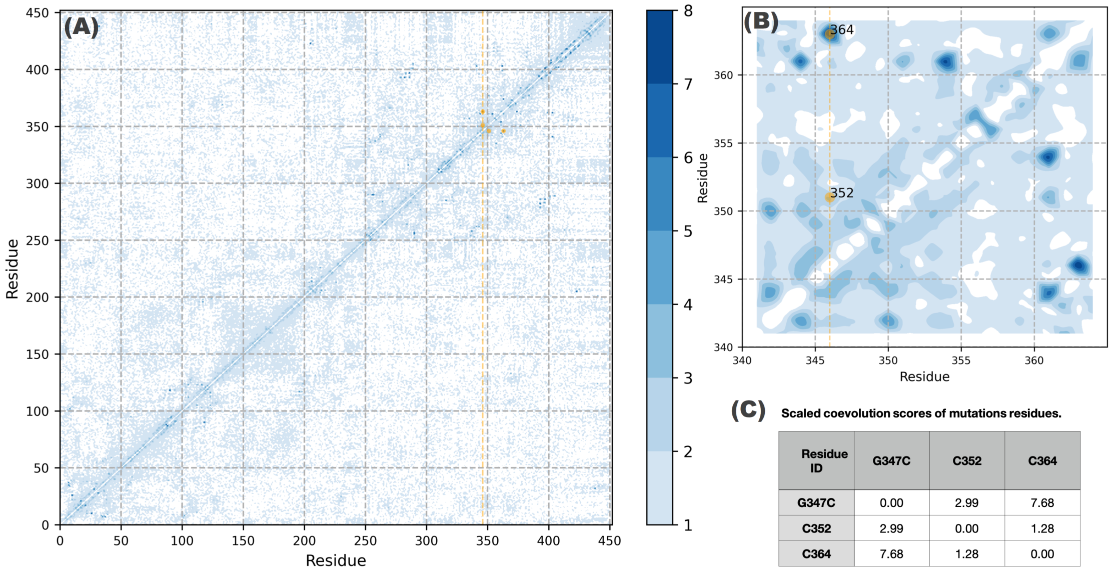

(C) Scaled coevolution scores of mutations residues.

Residue ID	G347C	C352	C364
G347C	0.00	2.99	7.68
C352	2.99	0.00	1.28
C364	7.68	1.28	0.00

Figure S7: Scaled coevolution score matrix for UDP-glycosyltransferase 76D1 highlighting (A) full length sequence and (B) residues 340-365. Residue pairs with non-significant score (<1) are shown in white. Residue 347 is shown by an orange line and its pairings with 352 and 364 are highlighted by orange circles; (C) Pairwise coevolution scores between residue pairs. G347 shows strongest coevolution signal with C364. The maximum scaled coevolution score was 8.3
